# CONUT score as a predictor for anamorelin efficacy in patients with cancer cachexia receiving chemotherapy

**DOI:** 10.1186/s40780-024-00359-5

**Published:** 2024-07-10

**Authors:** Hironori Fujii, Akitaka Makiyama, Kayoko Nishimura, Hirotoshi Iihara, Chiemi Hirose, Koichi Ohata, Yunami Yamada, Daichi Watanabe, Itaru Yasufuku, Naoki Okumura, Yoshihiro Tanaka, Takao Takahashi, Ryo Kobayashi, Nobuhisa Matsuhashi, Akio Suzuki

**Affiliations:** 1 Department of Pharmacy, Gifu University Hospital, Gifu, Japan; 2 Cancer Center, Gifu University Hospital, Gifu, Japan; 3 Center for Nutrition Support and Infection Control, Gifu University Hospital, Gifu, Japan; 4 Innovative and Clinical Research Promotion Center, Gifu University Hospital, Gifu, Japan; 5 Department of Gastroenterological Surgery/Pediatric Surgery, Gifu University Graduate School of Medicine, Gifu, Japan; 6 Laboratory of Advanced Medical Pharmacy, Gifu Pharmaceutical University, Gifu, Japan

**Keywords:** Cancer cachexia, Anamorelin, CONUT score, Survival, Performance status

## Abstract

**Background:**

Anamorelin is expected to improve cancer cachexia by increasing lean body mass (LBM) due to increased appetite and protein synthesis. However, the effect of anamorelin on cancer cachexia in real-world practice is unclear. The purpose of this study was to evaluate the efficacy and safety of anamorelin and to identify predictors of efficacy on treatment with anamorelin.

**Methods:**

We retrospectively analyzed data from patients with cancer cachexia treated with chemotherapy between May 2021 and August 2022. Efficacy of anamorelin was evaluated using LBM, with “12-week sustained effective response” to anamorelin treatment defined as maintenance or an increase in LBM for 12 weeks. We examined factors associated with “12-week sustained effective response” to anamorelin treatment using a multivariable logistic model that included controlling nutritional status (CONUT) score, an objective assessment of nutritional disorders, and the modified Glasgow prognostic score (mGPS), which scores the cachexia status of cancer patients. To assess patient subjective quality of life (QOL) changes related to eating after starting anamorelin treatment, we used a questionnaire (QOL-ACD appetite-related items: Q8, 9, 11). Adverse events were evaluated in accordance with the Common Terminology Criteria for Adverse Events (CTCAE) Version 5.0.

**Results:**

On analysis of data from 40 patients, 23 patients showed a 12-week sustained effective response to anamorelin (57.5%). At 12 weeks, LBM significantly increased by 1.63 ± 3.73 kg (mean ± SD). Multivariable logistic analysis revealed that a low CONUT score was significantly associated with “12-week sustained effective response” to anamorelin treatment (adjusted odds ratio: 13.5, 95% confidence intervals: 2.2–84.2, *P* = 0.004). QOL assessment showed a trend toward increased appetite and enjoyment of meals after anamorelin initiation. Five patients (12.5%) had an increase in HbA1c of more than 1.0% during the 12 weeks after the start of anamorelin. No patient had QT interval prolongation or grade 3 or higher hepatic transaminase elevation.

**Conclusion:**

Anamorelin may maintain or increase LBM with tolerable safety in patients with cancer cachexia undergoing chemotherapy. A low CONUT score, despite meeting criteria for cancer cachexia, is suggested as a predictor for the efficacy of anamorelin, indicating that patients with a low CONUT score may benefit from early introduction of anamorelin.

## Background

Cancer cachexia is defined as “a multifactorial syndrome characterized by a persistent loss of skeletal muscle mass (with or without fat loss) that cannot be completely reversed by conventional nutritional therapy and progresses to functional impairment” [1]. The European Palliative Care Research Collaborative (EPCRC) diagnoses cachexia in patients who meet any of the following three criteria: (1) Weight loss > 5% over the past 6 months, (2) BMI < 20 and any degree of weight loss > 2%, (3) Sarcopenia and any degree of weight loss > 2% [1]. The development of cachexia involves inflammatory cytokines produced as a biological response and various factors produced by tumor cells, which together lead to abnormal energy metabolism and the degradation of skeletal muscle [2]. The main symptoms are weight loss, loss of skeletal muscle mass, and anorexia, and it is this last factor – anorexia - which reduces quality of life (QOL).

Cancer cachexia is classified into three stages: precachexia, cachexia, and refractory cachexia [1]. Kimura et al. reported that the prognosis of patients with advanced non-small cell lung cancer complicated with cachexia is worse than that of patients without cachexia, no matter what stage of cachexia develops during the course of the disease [3]. In cancer patients, weight loss is associated with a variety of risks, including increased side effects during chemotherapy, fewer cycles of chemotherapy, less effective chemotherapy and radiation therapy, increased risk of surgery, and ultimately, decreased survival [4–8]. Furthermore, the efficacy of these combined therapies, including immune checkpoint inhibitors and cytotoxic anticancer drugs, is reportedly decreased in patients with cancer cachexia [9–12]. Thus, cancer cachexia is closely related to the efficacy of treatment, and an improvement of cancer cachexia is considered essential.

Although the European Palliative Care Research Collaborative (EPCRC) recommends intervention from the pre-cachexia stage [1], no standard treatment strategy to improve metabolic abnormalities in cancer cachexia has yet been established. Rather, a combination of pharmacotherapy and exercise therapy is currently considered effective in addressing the treatable factors of cachexia [13].

Anamorelin is an oral drug with ghrelin-like effects. In preclinical studies (in vitro and in vivo studies), anamorelin was shown to be a potent and highly specific ghrelin receptor (growth hormone secretagogue receptor type 1a) agonist with significant effects in stimulating appetite, increasing food intake and weight, and stimulating GH secretion [14]. GH promotes the secretion of insulin-like growth factor-1 (IGF-1) from the liver, and IGF-1 increases muscle mass. Anamorelin has high affinity (0.70 nM) for ghrelin receptors, a level which is slightly lower than that of natural ghrelin, and has no antagonist properties [14]. An in vitro report including anamorelin revealed that access of ghrelin ligands to the brain, particularly to the reward areas, is important for eliciting more potent appetite stimulant effects [15].

Currently, however, clinical evidence for the efficacy of treatment with anamorelin is insufficient. In particular, it is unclear which type of patient would most benefit from this agent.

We have focused on controlling nutritional status (CONUT) score and the modified Glasgow prognostic score (mGPS). The CONUT score is an index calculated from serum albumin concentration, total peripheral lymphocyte count, and total cholesterol concentration. CONUT scores are associated with sarcopenia and physical function in elderly patients with colorectal cancer [16]. Patients with cancer cachexia have been reported to have significantly lower total cholesterol compared to patients without cancer cachexia or non-cancer patients [17]. Accordingly, total cholesterol levels might indicate the stage of cachexia. Nevertheless, no study to date has described the use of CONUT score to predict the effectiveness of cancer cachexia treatment.

The mGPS, consisting of C-reactive protein (CRP) and albumin, is one of the most extensively validated prognostic factors in some cancer types [18–21].

Here, we evaluated the efficacy of anamorelin in clinical practice and identified patients who would benefit from anamorelin by using the CONUT score and mGPS.

## Methods

### Patients

This study was conducted under a retrospective observational design using data obtained from patient electronic medical records at our hospital. The study population consisted of patients with cancer cachexia who were started on anamorelin at Gifu University Hospital between May 2021 and August 2022. We excluded patients whose LBM was not evaluated before starting anamorelin.

### Criteria for administration of anamorelin

In our institution, anamorelin is prescribed in the outpatient chemotherapy unit if the physician confirms that the patient has had weight loss of 5% or more and anorexia within 6 months, and two or more of the following: (1) fatigue or malaise; (2) generalized muscle weakness; and (3) CRP > 0.5 mg/dL, hemoglobin < 12 g/dL, or albumin < 3.2 g/dL. If a pharmacist confirms the above criteria, they propose the prescription of anamorelin to the physician, who then prescribes anamorelin.

### Efficacy and safety of anamorelin

Patients included in the study received nutritional guidance and body composition assessment by dietitians before anamorelin was started. The body composition assessment was performed by dietitians using the direct segmental multi-frequency bioelectrical impedance analysis method (DSM-BIA) with InBody [22]. Electrocardiogram, blood glucose, and liver function marker measurements were also used to assess side effects. The evaluations included assessment of body composition, QOL, and side effects of anamorelin. They were performed every 3–4 weeks, following which the physician decided whether to continue anamorelin. The primary study outcome was “12-week sustained effective response” to anamorelin treatment, defined as maintenance or an increase in LBM for 12 weeks.

To evaluate the safety of anamorelin, we investigated the elevation of hepatic transaminases above grade 3, QT prolongation, and onset or exacerbation of diabetes mellitus. These adverse events were evaluated in accordance with the Common Terminology Criteria for Adverse Events (CTCAE) Version 5.0. The exacerbation of diabetes was defined as an increase in HbA1c of 1% or more from baseline. Every 3–4 weeks after starting anamorelin, the physician decided whether to continue anamorelin or not based on the evaluation of changes in LBM, improvement in anorexia and adverse events.

### Assessment of quality of life

QOL was assessed using a QOL questionnaire for cancer patients treated with anticancer drugs (QOL-ACD) [23], and performed by pharmacists or nurses. Patient quality of life was assessed using QOL-ACD Q8, Q9, and Q11, namely “Did you have a good appetite?” for Q8, “Did you enjoy your meals?” for Q9, and “Did you lose any weight?” for Q11. Patients answered using a 5-point scale for each of these 3 questions. Q8 and Q9 defined responses of “1” and “5” as “not at all” and “very much”, respectively, while Q11 defined responses of “1” as “no, I have instead gained weight” and “5” as “Yes”.

### Statistical analyses

Statistical analyses were conducted using IBM SPSS version 22 (IBM Japan Ltd., Tokyo, Japan), R software version 3.5.1 (www.r-project.org) and GraphPad Prism version 6.0 (GraphPad Software, San Diego, CA, USA). *P*-values less than 0.05 were considered significant. Patient characteristics were described as medians with 25th and 75th percentiles for continuous variables, and by frequency and percentage for categorical variables.

The percentage of patients who obtained a response was compared for PS (0, 1, 2), CONUT score (0–1: normal, 2–4: mild, 5–8: moderate, 8<: severe), and mGPS (0,1,2), respectively.

We showed mean changes from baseline values (± SD) in LBM, body weight, and skeletal muscle mass at each of weeks 3–4, weeks 8–9, and week 12. Comparisons of means between two or more corresponding groups were performed using repeated measures ANOVA. Results for the three QOL-ACD questions (Q8, 9, and 11) are shown as the percentage of patients with each response for each timing, i.e., at baseline, weeks 3–4, weeks 8–9, and week 12, respectively. We performed a Cochran Q-test statistical analysis with each QOL question as a categorical variable divided into level ≤ 3 and level > 3, respectively.

We performed multivariable logistic regression analyses to evaluate the associations between response to anamorelin and mGPS, as well as between response to anamorelin and CONUT scores [16–21]. First, both mGPS and CONUT scores were incorporated into the regression model by the forced entry method. Second, either mGPS or CONUT score was incorporated into the regression model along with one potential confounder at a time (PS, age, gender, BMI, gastric cancer) that may impact the 12-week sustained effective response to anamorelin. The reliability of the regression model was internally validated via a bootstrap method by measuring overfitting quantified by optimism parameter in a calibration plot. Bootstrap validation was performed with one hundred fifty resamples. Variance Inflation Factor (VIF) was calculated to check the multicollinearity between CONUT score and mGPS. To adjust for confounding, we performed logistic analyses by including one factor (PS, age, gender, BMI, gastric cancer) at a time in the CONUT score and mGPS models that may have an impact on 12-week sustained effective response to anamorelin.

## Results

### Patient demographics

Of the 55 patients who started anamorelin, 15 patients met the exclusion criterion of no LBM measurement before the initiation of anamorelin, leaving 40 patients for inclusion in the analysis. Patient background is shown in Table 1. Of the 40 patients, 30 (75%) were male and 10 (25%) were female. By cancer type, gastric, pancreatic, colorectal, and lung cancers accounted for 23, 9, 6, and 2 patients, respectively. Median LBM was 42.2 kg (interquartile range [IQR]: 36.1–47.1), and performance status (PS; 0, 1, 2) was 6, 29, and 5, respectively. All patients were cStage IV. In this study, none of the patients took drugs that stimulate ghrelin release, such as olanzapine or rikkunshito, during the period of anamorelin administration. Nutritional therapy included the introduction of high-protein diets and nutritional supplements at the discretion of the dietitian. Data on adherence to nutritional therapy was difficult to obtain because the patients in this study were undergoing outpatient chemotherapy, and it is difficult in real-world practice to collect strict data on adherence to nutritional therapies during an interview every few weeks. Further, exercise therapy was not provided to patients.

**Table 1:** Patient characteristics

Number of patients (male/female)	40	(30/10)
Age, median (range)	72	(48–83)
Performance status (0/1/2)	6 / 29 / 5	
Cancer type (gastric/pancreatic/colorectal/lung)	23 / 9 / 6 / 2	
Height (cm)	164.3	(158.0–169.2)
Weight (kg)	51.1	(43.6–58.1)
Body Mass Index	18.5	(16.9–20.6)
Lean body weight (kg)	42.2	(36.1–47.1)
Skeletal muscle mass (kg)	22.2	(19.1–24.8)
Total protein (mg/dL)	6	(5.6–6.3)
Albumin (mg/dL)	3.2	(2.9–3.5)
Pre-albumin (mg/dL)	15	(10.2–18.8)
Retinol-binding protein (mg/dL)	2	(1.6–2.6)
Transferrin (mg/dL)	184	(157.5–214)
C-reactive protein (CRP, mg/dL)	0.93	(0.3–2.0)
Hemoglobin (g/dL)	10.45	(9.6–12.0)
mGPS (0/1/2)	6 / 12 / 22	
CONUT score (0–1/2–4/5–8/8<)	5 / 13 / 20 / 2	
Treatment regimen		
Oxaliplatin + fluoropyrimidines ± nivolumab/trastuzumab	9	42.5%
Nivolumab or pembrolizumab	6	15.0%
FOLFIRINOX/FOLFOXIRI	6	15.0%
Gemcitabine + Nab-PTX	4	10.0%
Ramcirumab + paclitaxel/Nab-PTX	2	5.0%
Docetaxel	1	2.5%
Encorafenib + binimetinib + cetuximab	1	2.5%
Bevacizumab + TAS-102	1	2.5%
Carboplatin + irinotecan	1	2.5%
Trastuzumab emtansine	1	2.5%

### Efficacy and safety of anamorelin

**Fig. 1:**
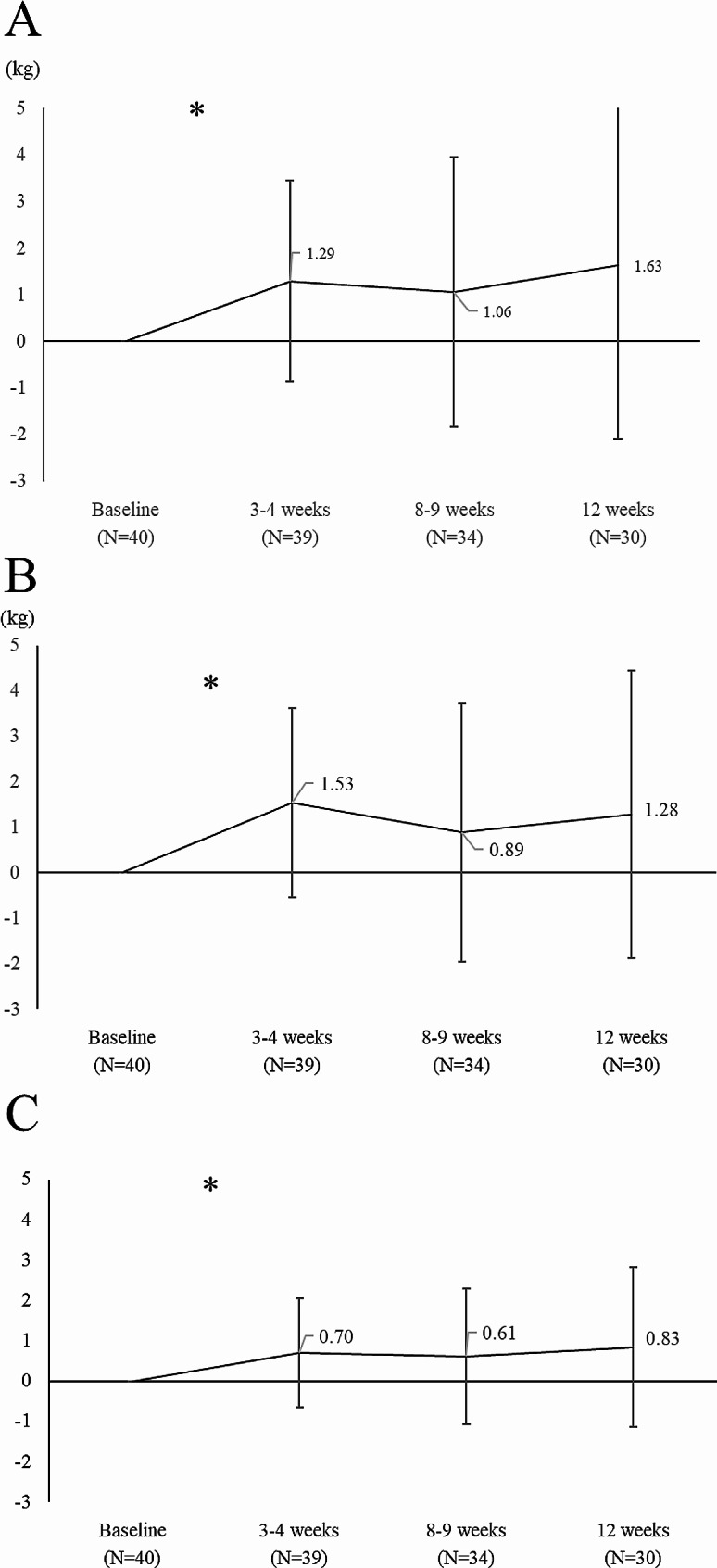
Change from baseline in (A) lean body mass, (B) body weight and (C) skeletal muscle mass after starting anamorelin treatment. *: *P* < 0.05 tested by repeated measures ANOVA

Twenty-three patients showed a response to anamorelin (57.5%). Changes in LBM, body weight, and skeletal muscle mass after anamorelin initiation over the 12-week course are shown in Fig. 1. The mean change (± SD) in LBM at weeks 3–4, 8–9, and 12 after anamorelin was 1.29 ± 2.16 kg, 1.06 ± 2.90 kg, and 1.63 ± 3.73 kg, respectively, a significant increase (*P* < 0.05). Changes in body weight and skeletal muscle mass were also significantly elevated at all points. Five patients (12.5%) had an increase in HbA1c of more than 1.0% during the 12 weeks after the start of anamorelin. No patient had QT interval prolongation or Grade 3 or higher hepatic transaminases elevation.

### Change in QOL-ACD (Q8, 9, 11) after the start of anamorelin

**Fig. 2:**
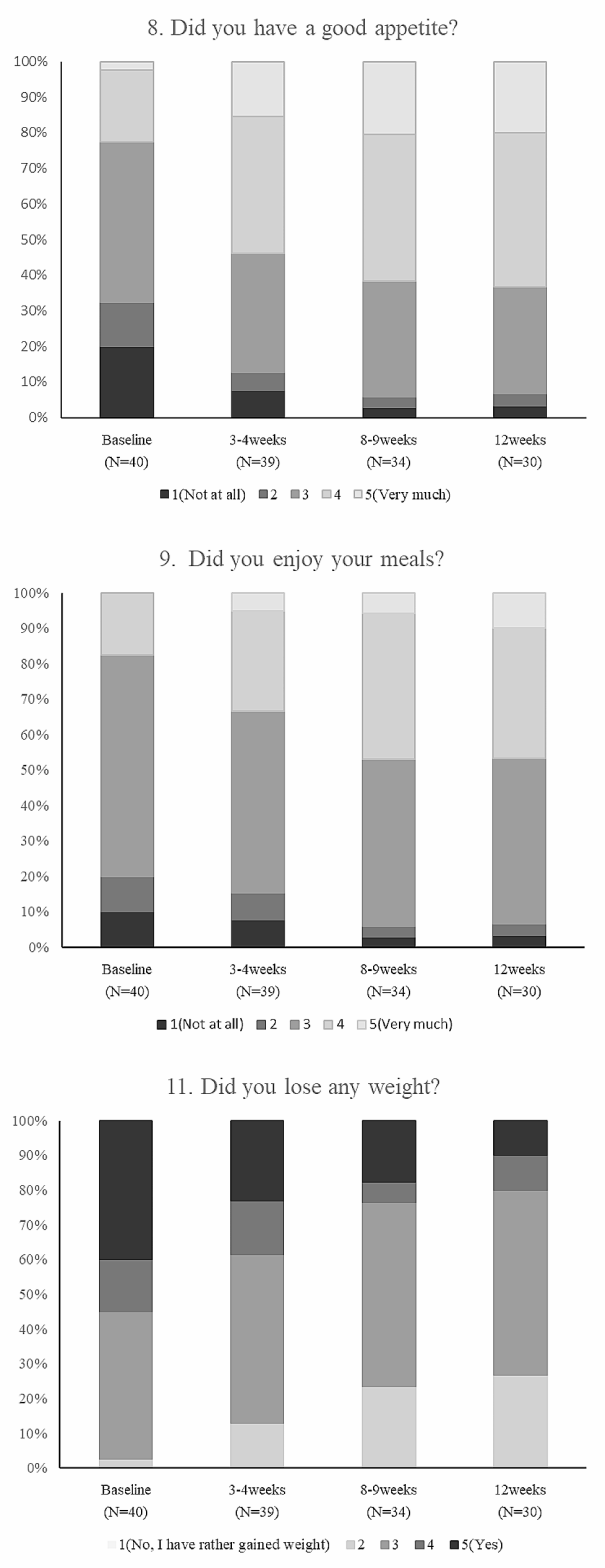
Change from baseline in QOL-ACD (Q8, 9, 11) after starting anamorelin treatment

We evaluated changes in the proportion of patients scoring in each of the five scores of the QOL-ACD questionnaire after starting anamorelin treatment (Fig. 2). The proportion of patients scoring at level 3 or lower gradually declined for Q8 (baseline: 77.5%, week 3–4: 46.2%, week 8–9: 38.2%, week 12: 36.7%) and Q9 (baseline: 82.5%, week 3–4: 66.7%, week 8–9: 52.9%, and week 12: 53.3%). In addition, for Q11, the proportion of patients scoring at level 3 or higher also gradually declined after the start of anamorelin (baseline: 97.5%, week 3–4: 87.2%, week 8–9: 76.5%, week 12: 73.3%). Cochran Q test results showed a significant increase in the percentage of scores > 3 for questions 8 (*P* = 0.036) and 9 (*P* = 0.009), and a significant decrease in the percentage of scores > 3 for question 11(*P* < 0.001).

### Determinants of a 12-week sustained effective response to anamorelin treatment

**Fig. 3:**
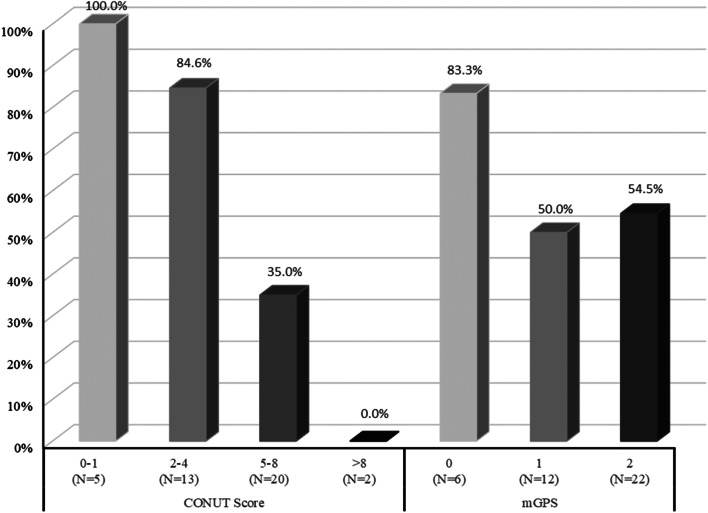
Percentage of patients with a response to anamorelin by each factor. “With a response to anamorelin” means “with maintained or increased lean body mass”

Figure 3 shows the percentage of patients who responded to anamorelin treatment in each group by CONUT score (0–1, 2–4, 5–8, > 8) and mGPS (0, 1, 2) factors. The results show that significantly more patients had a 12-week sustained effective response to anamorelin treatment in the CONUT 0–1 and CONUT 2–4 groups than in the CONUT 5–8 and CONUT > 8 groups. The mGPS0 group had a higher response rate to anamorelin than the mGPS1 and mGPS2 groups. As shown in Table 2, a low CONUT score (< 5) was a significant independent predictor for patients with a 12-week sustained effective response to anamorelin treatment (OR: 13.5, 95% CI: 2.2–84.2, *P* = 0.004). As shown in Table 3, CONUT scores significantly affected 12-week sustained effective response to anamorelin in all factors, while mGPS did not affect it in any factor.

**Table 2:** Multivariable logistic analysis of predictors of 12-week sustained effective response to anamorelin

Factor	OR (95%CI)	*P*-Value
mGPS < 2	0.63 (0.13–3.04)	0.562
CONUT score < 5	13.5 (2.2–84.2)	0.004

Abbreviations: CI, confidence interval; OR, odds ratio; CONUT score, controlling nutritional status score; mGPS, modified Glasgow prognostic score; 12-week sustained effective response, maintaining or increasing lean body mass for 12 weeks after start of anamorelin

**Table 3:** Associations of CONUT score and mGPS with anamorelin response, adjusted for confounding factors in bivariable logistic regression analysis

Adjustment variables	CONUT score			mGPS		
	OR	95%CI	*P*-Value	OR	95%CI	*P*-Value
PS	0.09	0.02–0.51	0.007	0.78	0.21–2.93	0.716
Age	0.09	0.02–0.48	0.005	0.77	0.22–2.77	0.694
Female	0.08	0.02–0.47	0.005	0.77	0.21–2.80	0.695
BMI	0.08	0.01–0.44	0.004	0.72	0.20–2.64	0.621
Gastric cancer	0.08	0.15–0.46	0.004	0.77	0.22–2.76	0.688

Logistic regression analysis was performed on either CONUT score or mGPS along with one of the adjustment variables each. Abbreviations: CI, confidence interval; OR, odds ratio; PS, performance status; BMI, body mass index; CONUT score, controlling nutritional status score; mGPS, modified Glasgow prognostic score

## Discussion

We evaluated the efficacy and safety of anamorelin in real-world clinical practice and identified predictors of efficacy of treatment with anamorelin. After starting anamorelin, LBM increased significantly, and QOL assessment showed improved appetite. Further, a low CONUT score (< 5) was identified as a predictor of efficacy of the treatment with anamorelin. On the other hand, a few patients developed new onset or exacerbation of diabetes after starting anamorelin. These results suggest that active anamorelin administration may be recommended for patients with anamorelin indications and low CONUT scores.

Previous studies included two international and one Japanese randomized, double-blind, placebo-controlled trials in patients with inoperable stage III or IV non-small cell lung cancer with cachexia. The two international phase III trials (ROMANA1, ROMANA2) compared anamorelin 100 mg with placebo at 93 centers in 19 countries and reported increases in LBM of 0.99 kg and 0.65 kg, respectively, after 12 weeks of anamorelin treatment, with both increases being significantly higher than placebo [24]. In the Japanese phase III study (ONO-7643-04), LBM increased by 1.38 kg after 12 weeks of anamorelin treatment, which was also significantly higher than placebo [25]. In our study, the mean increased LBM change at 12 weeks after anamorelin initiation was 1.63 kg, similar to the results of these studies [24, 25].

Further, in the current study all 5 patients with CONUT 0–1 and 11 of the 13 patients with CONUT 2–4 had anamorelin responses, while only 7 of the 22 patients with CONUT ≥ 5 had anamorelin responses. In other words, the anamorelin response rates for CONUT < 5 and CONUT ≥ 5 were 88.9% (16/18) and 31.8% (7/22), respectively, clearly higher for CONUT < 5. Unfortunately, we could only include the CONUT score and mGPS in our multivariable logistic model as predictors of anamorelin efficacy to avoid overfitting due to small sample sizes. However, the results of our multivariable logistic model and the difference in the proportion of anamorelin responders also suggest that the CONUT score may be a useful predictor of response when considering starting anamorelin.

Iwai et al. compared baseline factors at initiation in patients with gastrointestinal cancer who did and did not respond to anamorelin [26]. They found that total protein, albumin, transferrin, and prognostic nutritional index were significantly higher in responders, whereas neutrophil/lymphocyte ratio and C-reactive protein/albumin ratio were significantly lower. This finding by Iwai et al. that the proportion of patients with a low nutritional status prior to initiation is higher in patients who do not respond to anamorelin supports our results. Takeda et al. compared changes in body weight and appetite after anamorelin treatment in two groups of pancreatic cancer patients with cachexia who were divided into moderate (5–10%) and severe (> 10%) weight loss groups [27]. Results showed that the moderate weight loss group (*N* = 8) gained significantly more weight than the severe weight loss group (*N* = 16). Although the number of patients in this study was small, it is possible that patients with a smaller amount of weight loss prior to anamorelin initiation may be more responsive to anamorelin.

Our results and the results of the two previous studies [26, 27] suggest that anamorelin may not be effective if the patient is extremely underweight or has low nutritional markers at initiation of the drug. In other words, cancer cachexia should be diagnosed in a timely manner in patients undergoing cancer chemotherapy, and anamorelin should be started early. Therefore, collaborative efforts by physicians, pharmacists, nurses, and dietitians to monitor anorexia and weight loss in patients may be vital to improving cancer cachexia and increasing LBM.

Several limitations of our study should be mentioned. First, it was conducted under a retrospective design and analysed data from a single center. Second, because the sample size was small and the number of factors included in the multivariable analysis was limited to avoid overfitting, consideration of confounding factors may have been insufficient. Third, improvement in patients’ physical abilities, such as grip strength and 6-minute walking distance, could not be assessed. Fourth, it was not possible to determine changes in dietary caloric intake.

## Conclusion

For patients with cancer cachexia, anamorelin was found to be highly effective and well tolerated. In addition, patients with cachexia but a low CONUT score may benefit from anamorelin. We consider that administration of anamorelin to patients with early-stage cancer cachexia, such as those with low CONUT scores, is appropriate.

## Data Availability

Not applicable.
